# Corneal neovascularization: updates on pathophysiology, investigations & management

**Published:** 2019

**Authors:** Zuhair Sharif, Walid Sharif

**Affiliations:** * Department of Ophthalmology, Royal Wolverhampton NHS Trust, Wolverhampton, United Kingdom; ** Department of Ophthalmology, University of Jordan Hospital, University of Jordan, Amman, Jordan

**Keywords:** cornea, neovascularization, Anti-VEGF, keratoplasty, gene therapy

## Abstract

**Objective.** Corneal neovascularization is a sight-threatening condition affecting more than 1.4 million people per year. Left untreated, it can lead to tissue scarring, oedema, lipid deposition, and persistent inflammation that may significantly affect visual prognosis and quality of life. The aim was to review the recent evidence relating to the pathophysiology, investigations and management of corneal neovascularization.

**Methods.** Literature review of prospective and retrospective studies, clinical trials and animal models relating to the pathophysiology, investigation and management of corneal neovascularization.

**Results.** Corneal neovascularization is characterized by the invasion of new blood vessels into the cornea caused by an imbalance between angiogenic and antiangiogenic factors that preserve corneal transparency as a result of various ocular insults and hypoxic injuries. Risk factors that have been implicated in the pathogenesis of the disease include contact lens wear, ocular surface disease, trauma, previous surgery and herpes. The results highlighted the current and future management modalities of corneal neovascularization, which includes corneal transplantation, laser - phototherapy, injections and topical treatment.

**Conclusion.** The future of corneal neovascularization is promising and this paper discusses the upcoming revolution in local gene therapy.

**Abbreviations.** HSK = herpes stromal keratitis, VEGF = vascular endothelial growth factor, VEGFR-1 = VEGF Receptor-1, FGF = Fibroblast growth factor, PDGF = Platelet-derived growth factor, IL-6 = interleukin-6, IL-7 = interleukin-7, IL-8 = interleukin-8, IRS-1 = insulin receptor substrate-1

## Introduction

A healthy cornea is a transparent, avascular tissue located anterior to the iris and the pupil. Maintaining transparency and avascularity is essential to preserve optimal vision as well as protect the eye against infections and structural damage. 

Abnormal new vessels can invade the corneal stroma from pre-existing pericorneal structures as a result of a disruption in the balance of angiogenic and antiangiogenic factors that normally preserve corneal transparency and subsequently lead to corneal neovascularization [**1**]. 

This occurs due to a wide variety of ocular insults, including infection, inflammation, ischemia, degeneration, trauma, and loss of the limbal stem cell barrier. Corneal pathologies that can lead to neovascularization include lipid keratopathy, corneal ulcers and scars, herpes eye disease, infectious keratitis, chemical burns, graft rejections and hypoxic insults from contact lens wear [**2**,**3**].

Corneal neovascularization is a sight-threatening condition and a growing public health concern. One study reported the estimated incidence rate of 1.4 million people per year, 12% of whom suffered subsequent loss of vision [**4**]. Moreover, 20% of corneal specimens taken from corneal transplant procedures have shown evidence of corneal neovascularization [**3**]. 

Currently, the treatment methodology depends on the state of maturation of the blood vessels at presentation. Established mature blood vessels do not require angiogenic growth factors, whereas immature blood vessels are dependent on them for proliferation, hence treatment is aimed at either removal of established vasculature or preventing neoangiogenesis [**5**]. The pathophysiology, investigations and various treatment options currently being undertaken as well as future therapeutic potentials are discussed in this review. 

## Methods

A PubMed review was performed, analyzing all publications from 1968 to 2018 concerning the topic “corneal neovascularization” (keywords: cornea, new vessels, neovascularization, angiogenesis, Anti-VEGF, penetrating keratoplasty, corneal transplant). Animal and human studies, published in English (full text), were included in this review and adhered to the Helsinki Declaration.

## Results

**Pathophysiology of Corneal Neovascularization**

The cornea is avascular in healthy individuals; however, under specific pathological circumstances, new capillaries can grow within the cornea. There are three categories of neovascularization based on severity: superficial neovascularization, vascular pannus and deep stromal vascularisation. The mechanisms of corneal neovascularization are observed in significant detail in animal models. It has been hypothesized from these models that corneal neovascularization commences as a result of insult or injury. It is known that a number of diseases and conditions can lead to the development of corneal neovascularization. The most common causes highlighted have been the wearing of contact lenses, inflammation of the eyelid, trauma, previous surgery and herpes [**3**]. 

When the cornea is damaged, the epithelial defects are normally healed by the corneal and limbal epithelium. The corneal limbus is located at the corneoscleral junction. The limbal epithelium is rich in stem cells with the capacity to differentiate from normal corneal epithelium. However, defects can occur, leading to these cells undergoing apoptosis and repaired abnormally by the conjunctival epithelium [**6**]. The problem arises as the conjunctival epithelium is rich in goblet cells and highly vascularized. Consequently, the resulting phenotype is optically inferior and leads to the deterioration of vision [**7**]. Furthermore, the process also leads to an irregular optical surface, weakened tensile strength, and incompetent barrier function.

Research suggests that IL-8 may also contribute to the manifestation of corneal neovascularization [**8**]. Strieter et al. demonstrated the relationship to be dose dependent [**9**]. High doses of 400ng/ cornea did not give rise to neovascularization, whereas doses in the range of 2-40ng/ cornea resulted in neovascularization. Furthermore, the study interestingly found regression of vascularity after 14 days, which suggested that IL-8 angiogenesis underwent dynamic modulation as it was observed in normal wound healing, suggesting a dynamic relationship between inflammation and wound healing. 

As previously mentioned, HSK can lead to the development of corneal neovascularization. HSK is classed as an immune mediated disease and due to the eye being immune privileged, has been considered a target tissue for HSK. It is believed that VEGF has a significant role in the development of corneal neovascularization as a result of HSK. It has been suggested that the presence of HSK leads to inhibition of VEGF receptor (sVEGFR-1) synthesis at a higher rate compared to VEGF resulting in a ratio imbalance between sVEGFR-1 and VEGF and therefore, the release of VEGF is accelerated to consequently cause angiogenesis [**10**]. Another source of VEGF is infected cells stimulating the production of VEGF as a result of IL-6 expression [**11**]. A similar relationship has been observed in response to infected cells expressing IL-7, which also stimulates nearby cells to release VEGF [**12**]. The excessive release of VEGF leads to the development of fragile blood vessels in the cornea.

Corneal neovascularization can have a significant negative impact on vision. The physical presence of the vessels blocking and diffracting light being the main mechanism of impact, with further influence from the deposition of lipids and proteins on the corneal stromal as well as damage to the structural integrity of the cornea. 

The hypothesized pathophysiology is extrapolated from animal studies therefore leaving some uncertainty as to whether the relationships described can be transferred to a human model. 

**Investigating Corneal Neovascularization in the Clinical Setting**

The cornea can be easily assessed in the clinical setting for examination. Slit lamp biomicroscopy can be used to determine changes to the cornea including topographical ones. Slit lamp aids are also particularly useful in determining the thickness of the cornea, which can also provide evidence of endothelial cell function. Diffuse illumination can be used to assess the cornea in terms of gross alterations, whereas indirect and retro-illumination can be used to detect lesions such as neovascularization. Neovascularization can occur very rapidly, and may be challenging to detect in early stages.

The risk of developing corneal neovascularization can be assessed during routine eye examinations. It has been proven that the condition is more prevalent amongst certain populations such as those who wear contact lenses. In these instances, such patients could be classed as high risk and screened at shorter intervals. This could significantly reduce the number of cases of vision loss associated with corneal neovascularization. 

For the techniques described so far, neovascularization is only observed in advanced cases when the condition is already well developed. In order to scientifically study the pathophysiology of the disease progression, it would be useful to obtain samples from the tissue to observe the expression of cell signaling molecules (such as VEGF, IL-6 and IL-7) and develop and monitor tests to detect such early factors in corneal neovascularization. 

**Current management of corneal neovascularization**

The treatment of corneal neovascularization is currently problematic. Corneal transplantation is at present the only successful universal treatment for this disease process. However, there are various treatment procedures that have an effect, such as topical treatments, injections and laser/ phototherapy. One therapeutic aim of these treatments is to initiate antiangiogenesis and stop the neoangiogenesis at early stages, whereas the other treatment modality aims to achieve angioregression by inducing reversion of immature vessels. 

***Corneal Transplantation***

Meta-analysis on 24,000 corneal grafts revealed that rejection of transplanted corneas is higher in patients with neovascularization. The analysis estimates that “presence of corneal neovascularization before surgery is 30% more likely that the transplant will fail, and more than doubles the risk of graft rejection”, in other words, the greater the neovascularization the higher risk of rejection [**13**]. Therefore, preparing and conditioning the vascularized cornea before transplantation is a hopeful potential therapeutic development.

***Treatment of Corneal Neovascularization- Laser/ Phototherapy***

Argon laser therapy for corneal neovascularization is the use of an argon laser beam, which passes through a clear cornea, but, when there are many vessels present, the haemoglobin (within the blood) absorbs the argon energy allowing corneal vessels to coagulate, which causes reversal of the corneal neovascularization [**14**]. Studies have shown its efficacy in regression of corneal neovascularization [**15**]. Photodynamic therapy involves a photosensitizing compound, light and oxygen. The compound is absorbed by the neovascular tissue and is activated through laser treatment, which causes free radicals to be released thus destroying the surrounding neovascular tissue and reversing corneal neovascularization [**16**]. It has been shown that photodynamic therapy is safe and has a high efficacy within humans; however, it is a very costly method of treatment as well as time consuming [**16**]. 

Both laser and phototherapy need further study to determine their efficacy when compared to other therapeutic strategies. Currently, safety concerns associated with laser therapy and the cost and time of phototherapy have been the negative issues coupled with this innovative treatment, resulting in the relatively low uptake in clinical practice. However, a recent study by Gerten et al. has shown that the combination therapy of bevacizumab with argon laser-therapy causes a marked decrease in corneal neovascularization, this being because the argon laser-induced coagulation closes the mature pathological blood vessels whilst the bevacizumab prevents new angiogenesis [**17**]. Therefore, the hope is that these therapies will be introduced as an adjunct and usage will increase.

***Injections***

As described previously, treatment can be administered in many ways, also including the administration of steroids and anti-VEGF agents through subconjunctival injections with similar efficacy to topical treatment. Petsogulu C et al. carried out a randomized control trial looking at the outcomes of subconjunctival bevacizumab in 30 eyes of 30 patients with corneal neovascularization [**18**]. 15 eyes randomized to receive 2.5mg/ 0.1ml subconjunctival injections and 15 eyes randomized to 0.9% saline. A standard therapy of preservative-free dexamethasone 0.1% drops four times a day was prescribed for all patients at baseline. 

The authors demonstrated a reduction in the mean area of corneal neovascularization by 36% in the 15 eyes that received bevacizumab compared with an increase of 90% in eyes that received saline placebo. After exclusion of one outlier with an exaggerated response, the placebo arm treated with topical dexamethasone 0.1% over 3 months showed only a 3% decrease in corneal neovascularization. 

Moreover, this method of treatment also allows the incorporation of gene therapy strategies. Gene therapy involves transferring therapeutic genes to the cornea through different vectors. There are safety concerns regarding viral vectors (adenoviruses, retroviruses or lentiviruses) but they are the most efficient in infecting the corneal epithelial cells with infection rates of 80-100%, allowing higher gene transfer rates compared to non-viral vectors [**19**]. The safety concerns include the potential of replication-deficient viral vectors such as adenoviruses and retroviruses to become replication-competent and pathogenic again. Furthermore, retroviral vectors randomly integrate their genome into host cells, which can lead to insertional mutagenesis to occur [**20**]. Gene therapies that influence angiogenic factors like VEGF have been investigated, for example Lai and colleagues transduced corneal epithelial cells with an adenovirus vector containing the VEGFR-1 gene in a rodent model and found that it successfully inhibited corneal neovascularization [**19**]. Gene therapy can also occur through intrasomal or subconjunctival injections or via electroporation and gene gun [**21**]. However, the use of viral vectors has the highest efficiency in transduction of genes [**22**]. Furthermore, when the adenovirus vector containing VEGFR-1 was subconjunctivally injected in a rat model of corneal neovascularization there was inhibition of the corneal neovascularization [**23**]. Likewise, when an adeno-associated viral vector containing the gene for human angiostatin (protein-angiogenesis inhibitor) was subconjunctivally injected in a rat model, the rats showing a significant decrease in corneal neovascularization [**24**]. Although gene therapy has shown promise in effectiveness there are still technical and safety issues which have to be overcome first [**25**].

***Topical Treatments***

Steroids and anti-VEGF agents are currently the mainstay initial treatment for corneal neovascularization [**25**]. Topical steroids such as cortisone, dexamethasone and prednisolone have all been shown to have an antiangiogenic effect and hence inhibit corneal neovascularization [**25**-**28**]. However, there are studies suggesting that steroids do not inhibit the development of corneal vascularisation [**29**]. This was however demonstrated in response to corneal neovascularization post chemical injury, with recent research suggesting positive outcomes in other scenarios [**30**]. Klintworth has shown that steroid use is most effective in suppressing angiogenesis when applied directly after or before corneal injury and if applied any later it has no effect on the development of corneal vascularisation [**29**]. It is thought that steroids work by inhibiting cell chemotaxis and by inhibiting pro-inflammatory cytokines like interleukin-1 and -6 [**31**]. They also cause lymphocytes to be killed and inhibit vascular dilation, which all amounts to their antiangiogenic effect [**31**]. The use of steroids (such as cortisone) in conjunction with heparin and cyclodextrins causes a greater antiangiogenic effect, this leading to the development of ‘angiostatic steroids’, which are thought to modulate collagen metabolism that can completely disintegrate the basement membrane of the blood vessels [**32**,**33**]. Heparin modulates the expression of anti-angiogenic and pro-angiogenic factors [**25**]. However, steroids have a considerable side effect profile with negative associations such as glaucoma and increased infection susceptibility due to their immune suppressive effect.

VEGF has been shown to be crucial in inflammatory corneal neovascularization through the rat experimental model [**34**]. The eye is a site which has ‘angiogenic privilege’ meaning it has a balance of pro-angiogenic and anti-angiogenic factors. Pro-angiogenic factors include VEGF, FGF and PDGF [**25**]. Selectively targeting these angiogenic growth factors is desirable over steroids due to their side effect profile and more selective action. Anti-VEGF drugs work by inhibiting VEGF which prevents new blood vessel formation through down regulation of endothelial cell proliferation. Bevacizumab is a humanized monoclonal antibody which binds to all VEGF isoforms [**35**].

Another study has shown that bevacizumab does have an immediate inhibitory effect on corneal neovascularization and inflammation, but the effects are very short-lived [**36**]. Lin and colleagues have similarly shown that early treatment with bevacizumab inhibits corneal neovascularization but late treatment does not display these features [**37**]. This shows that anti-VEGF therapy is not as effective in individuals who have mature blood vessels as they do not rely on pro-angiogenic factors [**37**]. Anti-VEGF treatment is important during active vessel growth which is characterized by the presence of immature blood vessels relying on pro-angiogenic factors for proliferation [**38**]. This is in line with the findings by Lin that anti-VEGF treatment (bevacizumab) is effective when used in early treatment of patients with corneal neovascularization [**37**]. Anti-VEGF treatment can have undesirable effects, including suppression of wound healing, corneal nerve regeneration and can systemically cause hypertension and cardiovascular disease [**25**]. Krizova showed that the use of bevacizumab is effective and very safe in treating active corneal neovascularization whether applied topically or given as a subconjunctival injection [**39**]. However, they also show that bevacizumab does not have the same effect on mature corneal neovascularization and this treatment does not cure the disorder.

## Discussion

***New Topical Therapeutic Advancement***

It has been shown that the activation of the IRS-1 proteins is vital in angiogenesis and it is overexpressed in corneal neovascularization sites [**40**]. Aganirsen is an antisense oligonucleotide, which inhibits the expression of IRS-1 mRNA, mRNA of interleukin-1beta and the mRNA of VEGF [**40**]. Cursiefen et al. conducted a phase 3 trial and found that topical administration of Aganirsen eye drops massively inhibits corneal neovascularization in patients with keratitis and that the need for future transplantation is not needed. They have also shown that Aganirsen is very safe and well tolerated in individuals [**41**].

Another novel advance in the treatment of corneal vascularisation is the use of matrix metalloproteinase inhibitors such as use of tetracyclines. Doxycycline is a tetracycline analogue, which has an antibiotic effect but is also a matrix metalloproteinase inhibitor. Matrix metalloproteinase are enzymes that degrade collagen, basement membranes and the extracellular matrix. Doxycycline also inhibits angiogenesis in a non-metalloproteinase-dependent mechanism, and has an anti-inflammatory effect [**42**]. Jovanovic and Nikolic have shown that the use of topical doxycycline on human corneal neovascularization is effective in reducing the neovascularization and effective in the healing process without any side effects [**43**]. Combination therapy of anti-VEGF agents, steroids and doxycycline have been investigated and have been shown to have higher efficiency in inhibiting corneal neovascularization compared to solitary use [**32**]. The theory behind combination therapy is that this method will target various mechanisms involved in maintaining corneal neovascularization and hence will be much more efficient in inhibiting the disease and its reoccurrence.

***New Injectable Therapeutic Advancements***

Pillai et al. first described the technique of fine needle diathermy, which involves using a needle to cauterize individual vessels; this method is effective in occluding mature vessels that are not dependent on angiogenic growth factors [**44**]. This technique has been modified through using an electrolysis needle that is much more flexible and precise [**45**]. Trikha et al. carried out a 5 year retrospective study on individuals who underwent fine needle diathermy and found that this treatment is safe and very effective in regressing corneal neovascularization [**46**]. This suggests that this method of treatment can be used in conjunction with anti-VEGF drugs to allow an angioregressive treatment of corneal neovascularization [**47**,**48**]. 

Gene therapy targeting VEGF has had successful results, for example Lai et al. have shown that an adenovirus vector carrying VEGFR genes caused regression of the corneal neovascularization [**49**]. Furthermore, when the adenovirus vector was subconjunctivally injected in a rat model, there was inhibition of the corneal neovascularization [**50**]. Likewise, when an adeno-associated viral vector containing the gene for human angiostatin (protein-angiogenesis inhibitor) was subconjunctivally injected in a rat model, the rats showed a significant decrease in corneal neovascularization [**51**]. Although gene therapy has shown promise in effectiveness, there are still current technical concerns.

## Conclusion

The ever-expanding knowledge of the mechanisms involved in corneal neovascularization are allowing different treatment options to be developed. Anti-VEGF drugs have been the centre of discussion as have matrix-metalloproteinase inhibitors. 

These methods of treatments for corneal neovascularization currently still depend on the blood vessel maturity stage. Therefore, local gene therapy may be a promising universal treatment of corneal neovascularization, with the hope that safety concerns can be allayed by continuing and impending research.

**Conflict of interests**

The authors declare no conflict of interests.

**Acknowledgements**

All authors have equal contribution. There are no financial or proprietary interests. 
